# Bone conduction implants and active middle ear implants for adults

**DOI:** 10.1177/00368504251328010

**Published:** 2025-03-17

**Authors:** Eleonor Koro, Jeremy Wales, Mimmi Werner

**Affiliations:** 1 Department of Clinical Sciences, Otorhinolaryngology, 8075 University of Umeå, Umeå, Sweden; 2 Department of Clinical Science, Intervention and Technology, 27106 Karolinska Institute, Stockholm, Sweden

**Keywords:** Bone conduction, bone-anchored hearing aid, transcutaneous bone conduction, percutaneous bone conduction, active middle ear implant

## Abstract

Hearing loss is the third most significant cause of disability globally and is associated with anxiety, depression, loneliness, and cognitive decline. For those unable to utilize conventional hearing aids due to conditions such as ear canal atresia, eczema, recurrent external otitis or extensive ear surgery, implantable hearing aids provide an alternative. This narrative review provides an overview of available bone conduction devices and active middle ear implants. The rapid advancements in implantable hearing aid technology necessitate ongoing education of healthcare professionals to enable informed patient decisions.

## Consensus

### Most people agree that:

- conventional hearing aids are the first choice for most forms of hearing loss
- reconstructive otosurgery is the first choice if the possibility of hearing aid independence exists
- implantable hearing aids may be considered for patients who have an inadequate effect from conventional hearing aids or are unable to use them
- patients with bilateral hearing loss benefit greatly from implantable hearing aids
- the incidence of skin infection is lower with transcutaneous implants than with percutaneous implants
- active middle ear implants generate a more authentic sound quality than bone conduction implants, but the surgery is more demanding and with a higher risk of complications
- a hearing solution for patients with unilateral hearing loss should always be considered

### Opinions differ regarding:

- which hearing solution is best for patients with single-sided deafness
- whether care givers should be more conservative with providing implantable hearing solutions for patients with unilateral hearing loss

## Introduction

Globally, hearing loss is the third most significant cause of disability.^1^ Hearing loss is associated with high levels of anxiety, depression, somatization, loneliness and dementia.^2,3^ In Sweden, approximately 1.5 million people suffer from hearing loss, but only about 500,000 currently have hearing aids. An additional 800,000 people in Sweden need hearing aids and globally this deficit is enormous.^4^

Conventional hearing aids can amplify sound intensity up to 70–80 dB and are the best option for most types of hearing loss. Ear canal eczema, recurrent external otitis, ear canal atresia or extensive ear surgery may mean that a conventional hearing aid cannot be worn. For these patients, as well as for patients with unilateral hearing loss (UHL) and single-sided deafness (SSD), implantable hearing aids may be an option. Bone conduction devices (BCDs) and active middle ear implants (AMEIs) provide audiologic benefits for patients with conductive or mixed hearing loss^5–7^ where AMEIs also provide audiologic benefits for mild to severe sensorineural hearing loss (SNHL).^8^ Although UHL was historically undertreated due to the misconception that the unaffected ear was sufficient for general speech development in early prelingual cases and adequate for functional hearing in adults, it is now widely acknowledged that addressing UHL is essential for optimizing outcomes in both children and adults. Most patients with UHL have impaired or absent binaural hearing leading to difficulties with sound localization, to perceive speech in noisy environments, and maintain spatial awareness.^9–11^ However, the best treatment for UHL is still under debate. BCDs reduce the head shadow effect and improve sound awareness on the affected side but do not restore binaural hearing.^12^

Furthermore, since bone-conducted sound is efficiently transmitted through the skull bone, interactions may occur within the normal-hearing cochlea between the air-borne signal and cross-stimulation generated by the bone conduction device (BCD).

A literature search was performed to conduct this narrative overview. Keywords such as “bone conduction”, “implantable hearing aids”, “middle ear implants”,“bone conduction implants”, “passive transcutaneous bone conduction implants”, “active transcutaneous bone conduction implants”, and “percutaneous bone conduction implants”, were used. This review is guided by the Scale for the Assessment of Narrative Review Articles (SANRA).^13^

The scope of this narrative review is to provide an updated overview of the BCDs and AMEIs available on the market today. The aim is to equip clinicians with an unbiased survey of available BCDs and AMEIs to assist patients in making informed choices regarding their hearing rehabilitation.

### Implantable hearing aids

**Figure 1.**
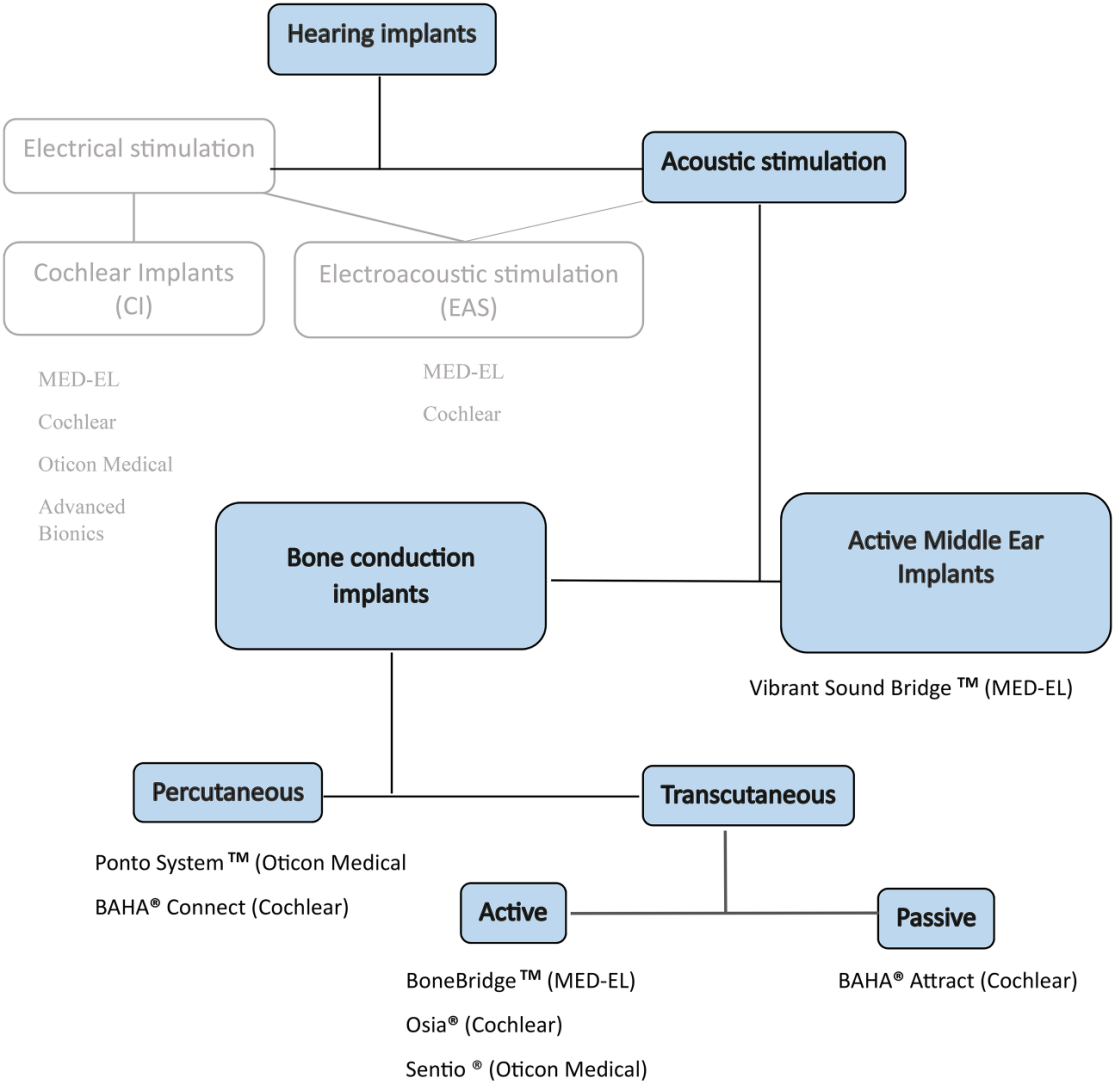
Categorization of hearing implants.

Implantable hearing aids stimulate electrically or acoustically. The implants that stimulate electrically include cochlear implants (CI) and electroacoustic stimulation (EAS). In EAS, as in CI, electrical stimulation is combined with acoustic stimulation where a conventional hearing aid amplifies the bass range. Acoustically stimulating implants include BCD and AMEI. BCD is divided into percutaneous with skin penetration, and transcutaneous with intact skin between the implant and sound processor. Transcutaneous BCDs are further categorized as passive or active. Passive transcutaneous BCDs generate vibrations in the external sound processor that are then transmitted through the skin to the implant. Active transcutaneous BCDs generate vibrations in the implant. AMEIs generate vibrations locally at the structures of the middle ear where they have been applied, i.e. ossicles or round window (see Figure 1).^14^

Bone conduction hearing occurs when vibrations are generated in the body's soft tissue and skull bone and are then continued via the skull bone directly to the inner ear. This results in a wave propagation along the basilar membrane and stimulation of the auditory nerve.^15^ The concept of bone conduction hearing was first documented by Girolamo Cardano in the sixteenth century.^16^ In the early twentieth century, conventional bone-conduction hearing aids were developed that consisted of a microphone, a sound processor, and a vibrator attached to either a pair of glasses or a headband. The first percutaneous BCD was developed in Gothenburg, and implanted in the first human in 1977.^17^

### For whom is an acoustic stimulating implant a treatment option?

#### BCD

BCD is a treatment option for people with bilateral conductive or mixed hearing loss, unilateral conductive or mixed hearing loss or unilateral deafness (Single-sided deafness (SSD)). BCDs provide a more improved hearing with a large air-bone gap (air-bone gap 30 dB or more) than conventional hearing aids for those with conductive or mixed hearing loss.^18^ Hearing thresholds for bone conduction should be better than 65 dB depending on the implant type. According to companies’ recommendations for UHL, the hearing ear should have a pure tone average for bone conduction at frequencies of 0.5, 1, 2 and 4 kHz (PTA4) equal to or better than 20 dB (Table 1), but this has been questioned.^19^ Therefore, all patients are required to sustain a trial period using a bone conducting hearing aid on an elastic band.

**Table 1.** Indications and contraindications for BCDs and AMEIs.

Implant	Ponto^TM^	Baha^®^ Connect	Baha^®^ Attract	Osia^®^	Bonbridge^TM^	Sentio Ti	Vibrant SoundBridge^TM^
Approval date	2009 CE approved	1985 CE approved	2013 CE approved	2019 CE approved	2012 CE approved	2024 CE approved	1998 CE approved
Indications	Unilateral hearing loss where air and bone conduction PTA 4 is ≤ 20 dB in the hearing ear. Conductive or combined hearing loss where PTA 4 for the bone conduction is ≤ 65 dB. No lower age limit, but if the skull bone is 2.5 mm or thinner, a two-stage operation is recommended.	Unilateral hearing loss where air and bone conduction PTA 4 is ≤ 20 dB in the hearing ear. Conductive or combined hearing loss where PTA 4 for the bone conduction is ≤ 65 dB. No lower age limit, but if the skull bone is 2.5 mm or thinner, a two-stage operation is recommended.	Unilateral hearing loss where air and bone conduction PTA 4 is ≤ 20 dB in the hearing ear. Conductive or combined hearing loss where PTA 4 for the bone conduction is ≤ 65 dB. No lower age limit, but if the skull bone is 2.5 mm or thinner, a two-stage operation is recommended. A skin thickness of at least 3 mm.	Unilateral hearing loss where air and bone conduction PTA 4 is ≤ 20 dB in the hearing ear. Conductive or combined hearing loss where PTA 4 for the bone conduction is ≤ 55 dB. No lower age limit with body weight over 7 kg with sufficient bone quality and quantity for successful implant placement.	Unilateral hearing loss where bone conduction PTA 4 is ≤ 20 dB in the hearing ear. Conductive or combined hearing loss where PTA 4 for the bone conduction is ≤ 45 dB. From 5 years of age.	Unilateral hearing loss where bone conduction PTA 4 is ≤ 20 dB in the hearing ear. Conductive or combined hearing loss where PTA 4 for the bone conduction is ≤ 45 dB. From 12 years of age.	Mild to severe sensorineural hearing loss with air conduction ≤ 65 dB in the low frequencies and ≤ 85 dB in the high frequencies. Conductive or combined hearing loss where the bone conduction is ≤ 45 dB in the low frequencies or ≤ 65 dB in the high frequencies. From 5 years of age.
Relative contraindications	Difficulty with hygiene around implantation. Difficult to handle the sound processor. Poor bone or skin quality such as radiation-damaged skin or skull.		Regular MRI examinations.				Active middle ear infections. Poor anatomical conditions that increase the risk of facilis damage, small middle ear space where the vibrator cannot fit.

CE = ConformitÉ EuropÉenne, PTA4 = Pure Tone Average of the frequencies 0.5, 1, 2, and 4 kHz, dB = decibel, MRI = Magnetic Resonance Imaging

#### AMEI

AMEI is indicated for people with sensorineural, conductive or combined hearing loss. The indication includes people with sensorineural hearing loss with air conduction better than 65 dB in low frequencies and better than 85 dB in high frequencies, as well as people with conductive or combined hearing loss where the bone conduction thresholds are better than 45 dB in low frequencies and better than 65 dB in high frequencies (Table 1). Preoperative computed tomography of the middle ear is recommended for assessment of risks and anatomical conditions for attaching the vibrator.

### Preoperative investigation

A multidisciplinary team with at least an audiologist and otologist is important to be able to offer the patient an individually adapted hearing solution.^20^ The choice between alternatives is complex and depends on several factors such as surgical, audiological and anatomical, and the patient's desires and expectations.

Step 1: Assessment of degree and type of hearing loss. As a basis for this assessment, tone and speech audiometry are performed, as well as, if necessary, an extended investigation such as stapedius reflexes and tympanometry.

Step 2: A trial period with a BCD on an elastic band, headband or self-adhesive adapter for four weeks. The aim is to evaluate the benefit of a BCD before surgery and to give the patient realistic expectations. An AMEI cannot be evaluated in a trial period.

Step 3: Assessment of whether the patient is a potential candidate for a BCD or AMEI and an evaluation of the test period. If there is an audiological indication and an interest in surgery in the patient, a supplementary hearing examination should be carried out as follows:

*Tone audiometry*: free-field measurements with warbler tones or narrow-band noise, with and without bone-conduction hearing aid.

*Speech audiometry:* with noise at a S/N of +4 dB and speech level of 65 dB or maximum speech perception at 65 dB, with and without bone conduction hearing aid.

A CT scan is recommended if the surgeon needs to evaluate the access route, the planned implant placement is over the mastoid or if there is a suspicion of reduced bone thickness or quality. It is essential in challenging anatomical conditions such as malformations, poor pneumatization, or after a canal wall down mastoidectomy. Often there is more than one choice for implant placement. MRI may be considered to rule out coexisting conditions with the understanding that postoperatively a large shadow will impair MRI scans.^21^

Step 4: An otologist, in consultation with the patient, decides on surgery and then makes a medical assessment and implant selection. Factors that should be discussed with the patient include, but are not limited to, skin penetration or intact skin, use of general anesthesia or local anesthesia, potential complications, and MRI compatibility. Adults are usually operated under local anesthesia for percutaneous BCD, and under general anesthesia for transcutaneous BCD and AMEI.

### Acoustic implants

#### Non-surgical bone conduction hearing aid

Non-surgical bone conduction hearing aids are attached behind the ear with an elastic band, headband or patch. These are used as part of the preoperative investigation while waiting for reconstructive ear surgery or as a hearing solution when surgery is not suitable. In non-developed countries, where implants, surgery and adequate postoperative care is limited, non-surgical bone conduction hearing aids can be used as a long-term solution.

#### Percutaneous BCD

The percutaneous BCD consists of a titanium implant anchored in the skull bone behind the outer ear as well as a spacer and a sound processor. The sound processor converts sound waves into vibrations that propagate through the skull bone to the cochlea. A significant advantage is the simple and quick operation that can be performed under local anesthesia. A disadvantage is that breaking through the skin increases the risk of infections and that continuous skin care around the abutment is required. For grading skin complications, Holger's index is often used with a scale from 0 to 4 where 0 represents the absence of skin irritation and 4 means that the abutment must be removed.^22^ An alternative is a recently developed grading scale called the Inflammation-Pain-Skin height/numbness scale (IPS) that can be used for both percutaneous and transcutaneous BCDs.^23^ A meta-analysis from 2013 that included adults and mixed age groups of adults and children, reported the incidence of Holger grade 2–4 skin reactions between 2.4% and 38.1%. Among the different studies in the meta-analysis, the total rate of implant loss due to failed osseointegration, trauma, infection or lack of benefit to the patient varied from 1.6% to 17.4%.^24^ A meta-analysis from 2020 reported the incidence of Holger grade 2–4 skin reactions to be 15%, and a survival rate of almost 98%, and thus, an implant loss of 2%. This indicates that with the more recent percutaneous BCDs and surgical techniques, the percutaneous-implant survival rate is high.^25^ On the Swedish market today, two percutaneous BCDs are available – Ponto^TM^ and Baha^®^ Connect, both of which are MR compatible up to 3 Tesla (Table 2).

**Table 2.** Sound processor specification for BCDs and AMEIs.

	Implant	Processor	Adaptation area Conductive or combined hearing loss	Adaptation area Single-sided deaf	Adaptation area Sensorineural hearing loss	Frequency range	MR compatibility
	Ponto^TM^ ₸	Ponto^TM^ 3	BC PTA4 ≤ 45 dB	Deaf in one ear and BC PTA4 ≤ 20 dB in the hearing ear		200–9500 Hz	3 Tesla
		Ponto^TM^ 3 Power	BC PTA4 ≤ 55 dB	Deaf in one ear and BC PTA4 ≤ 20 dB in the hearing ear		260–9600 Hz	3 Tesla
		Ponto^TM^ 3 Superpower	BC PTA4 ≤ 65 dB	Deaf in one ear and BC PTA4 ≤ 20 dB in the hearing ear		260–9600 Hz	3 Tesla
Percutaneous		Ponto^TM^ 4	BC PTA4 ≤ 45 dB	Deaf in one ear and BC PTA4 ≤ 20 dB in the hearing ear		200–9500 Hz	3 Tesla
	Baha^®^ Connect£	Baha^®^ 5	BC PTA4 ≤ 45 dB	Deaf in one ear and BC PTA4 ≤ 20 dB in the hearing ear		250–7000 Hz	3 Tesla
		Baha^®^ 5 Power	BC PTA4 ≤ 55 dB	Deaf in one ear and BC PTA4 ≤ 20 dB in the hearing ear		250–7000 Hz	3 Tesla
		Baha^®^ 5 SuperPower	BC PTA4 ≤ 65 dB	Deaf in one ear and BC PTA4 ≤ 20 dB in the hearing ear		250–7000 Hz	3 Tesla
		Baha^®^ 6 Max	BC PTA4 ≤ 55 dB	Deaf in one ear and BC PTA4 ≤ 20 dB in the hearing ear		200–9700 Hz	3 Tesla
Passive Transcutaneous	Baha ^®^ Attract £	Baha^®^ 5	BC PTA4 ≤ 45 dB	Deaf in one ear and BC PTA4 ≤ 20 dB in the hearing ear		250–6300 Hz	1.5 Tesla
		Baha^®^ 5 Power	BC PTA4 ≤ 55 dB	Deaf in one ear and BC PTA4 ≤ 20 dB in the hearing ear		250–7000 Hz	1.5 Tesla
		Baha^®^ 5 SuperPower	BC PTA4 ≤ 65 dB	Deaf in one ear and BC PTA4 ≤ 20 dB in the hearing ear		250–7000 Hz	1.5 Tesla
		Baha^®^ 6 Max	BC PTA4 ≤ 55 dB	Deaf in one ear and BC PTA4 ≤ 20 dB in the hearing ear		200–9250 Hz	1.5 Tesla
Active Transcutaneous	Osia^®^ £	Osia^®^ 2	BC PTA4 ≤ 55 dB	Deaf in one ear and BC PTA4 ≤ 20 dB in the hearing ear		400–7000 Hz	3 Tesla
	Bonebridge^TM^ ¥	Samba^TM^ 2	BC PTA4 ≤ 45 dB	Deaf in one ear and BC PTA4 ≤ 20 dB in the hearing ear		250–8000 Hz	1.5 Tesla
	Sentio Ti	Sentio 1 Mini	BC PTA4 ≤ 45 dB	Deaf in one ear and BC PTA4 ≤ 20 dB in the hearing ear		200–9500 Hz	1.5 Tesla
Active middle ear implants	Vibrant SoundBridge^TM^ ¥	Samba^TM^ 2	BC ≤ 45 dB vid 0.5 kHz och ≤ 65 dB vid 2-4 kHz		AC ≤ 65 dB vid 0.5 kHz och ≤ 85 dB vid 3-6 kHz	250–8000 Hz	1.5 Tesla

BC = Bone conduction, AC = air conduction, PTA4 = Pure Tone Average for the frequencies 0.5, 1, 2, and 4 kHz, ╤ = Oticon Medical AB, Askim, Sweden. £ = Cochlear™, Sydney, Australia. ¥ = MED-EL, Innsbruck, Austria.

#### Passive transcutaneous BCD

Passive transcutaneous BCDs generate vibrations in the external sound processor that are then transmitted through the skin to the implant. The Baha^®^ Attract is a passive transcutaneous BCD that is on the Swedish market. It consists of a bone-anchored titanium implant (BI300) that is anchored directly to the skull bone behind the outer ear and a magnet that is attached to this implant. The sound processor is connected to an external magnet and the two magnets are held together with intact skin between them. The sound processor converts incoming sounds into vibrations that are propagated via the skin and underlying tissue to the titanium implant and on to the cochlea. With passive transcutaneous BCDs the skin is intact, so the risk of infection is lower than with percutaneous BCDs, and daily cleaning is not needed. Moreover, passive transcutaneous BCDs are perceived as cosmetically advantageous compared to percutaneous BCDs. Although the rate of skin complications is lower than with percutaneous BCD, the force required to hold the two magnets together can cause pain and irritation. A systematic review reported an incidence of minor skin problems that healed on their own or after switching to a weaker magnet of 13.1%. In the same review, the incidence of major skin problems such as postoperative seroma, hematoma, wound infection or skin lesions that required active intervention was 5.2%.^26^

For passive transcutaneous BCDs when vibrations travel through the skin, sound transmission is attenuated with a loss of approximately 5 dB at 1 kHz and up to 25 dB at 6–8 kHz compared to percutaneous and active transcutaneous BCDs.^27^ An advantage of the Baha^®^ Attract is that if it does not provide sufficient amplification, it can be converted to a percutaneous BCD via a minor operation where the magnet is replaced with a spacer. Passive transcutaneous BCDs have been shown to achieve comparable hearing benefits as non-surgical bone conduction hearing aids,^28,29^ for this reason, there is a discussion about whether clinicians should become more conservative with providing invasive passive transcutaneous systems. The Baha^®^ Attract is MRI compatible at 1.5 Tesla with the internal magnet in place (Table 2). A large area of artifact will be present on the MRI that is significantly greater than that that occurs with percutaneous BCD.

#### Active transcutaneous BCD

Active transcutaneous BCDs generate vibrations in the subcutaneous implant and not in the external sound processor. There are three active transcutaneous BCDs on the Swedish market – the Bonebridge^TM^ (Med-El, Innsbruck, Austria), the Osia^®^ (Cochlear BAS, Gothenburg, Sweden) and Sentio Ti (Oticon Medical). An external sound processor converts incoming sounds into digital signals that are transmitted to an implanted part where the signals are in turn converted into vibrations via either electromagnetic (Bonebridge^TM^ and Sentio Ti) or piezoelectric (Osia^®^) stimulation. The sound processor and the implanted part are separated by intact skin and held together by a magnet on either side. Since the vibrations that are to be propagated via the skull bone are created in the internal implanted part, there is no attenuation during the transmission as with passive transcutaneous BCDs and a lower magnetic strength can be used. The operation is usually performed under general anesthesia and takes about 1 h; and it is somewhat more demanding than for percutaneous BCD. The vibrator on the Bonebridge^TM^ is drilled 4.5 mm into the mastoid and attached with two screws. The Osia^®^ consists of two implanted parts where the Cochlear BI300 implant is applied to the skull bone and then the OSI300 implant is screwed onto this. Sentio is drilled up to maximum 3 mm into the mastoid and attached with a fixation band and two screws. Two systematic reviews regarding the Bonebridge^TM^ report 1.7–5.7% serious complications, where a serious complication was defined as a complication that required revision surgery or explantation.^30,31^ Corresponding studies are lacking for the Osia^®^ and Senti Ti. Anecdotal reports indicate that some patients report cosmetic problems because the OSIA actuator is raised from the skull above the titanium implant. This concern is more pronounced in pediatric patients compared to adults. Both Bonegridge^TM^ and Snetio Ti are MRI compatible at 1.5 Tesla with the internal magnet in place and for the Osia(OSI300)^®^ at 3 Tesla (Table 2).

#### AMEI

Currently, there is one approved active middle ear implant (AMEI) available in Sweden – the Vibrant SoundBridge^TM^ (MED-EL, Innsbruck, Austria). It consists of an external sound processor and an internal implant that is attached to the skull bone behind the ear. The components are held together by means of two magnets with intact skin between them. From the implant, there is a lead to a vibrator (floating mass transducer, FMT) that attaches to the incus short or long processes, the stapes head or plate, or the round window via a variety of couplers. The sound is captured by the sound processor and converted into a signal that is sent on to the FMT where it is converted via electromagnetic stimulation into vibrations. Most often, a computed tomography scan is performed preoperatively to map the anatomy of the middle ear. The operation is performed under general anesthesia and requires drilling of the mastoid and takes about 2.5 h. The Vibrant SoundBridge^TM^ is MR compatible at 1.5 Tesla (Table 2). AMEI, compared to BCD, is considered to deliver a more natural sound because it is transmitted through the ossicular chain or the round window. The different couplers include the RW-soft or RW coupler for the round window, the Incus LP-coupler or Symphonix-Coupler for the incus long process, the Incus SP-coupler for the short process, Vibroplasty CliP-Coupler, SH-coupler or Vibroplasty bell-coupler for the stapes super structure, and the OW (oval window)-coupler for the stapes footplate.^32–36^ There are also a few studies showing improved directional hearing and speech in noise when AMEI provides side-specific stimulation.^37,38^ The effect of the sound transmission varies depending on where and how the connection to the bone is made. The most common complication of AMEI is taste disturbance due to chorda tympani damage, but damage to the facial nerve also occurs.^39^

### Postoperatively

Healing control takes place approximately 1 week after surgery.

Approximately 4 weeks postoperatively, the hearing test and fitting of the external sound processor are done. In the case of thin or radiation-damaged bone or skin, the time between surgery and fitting should be extended.

One should book a follow-up visit to the audiologist to evaluate the effect of the implant with sound field measurements with tone and speech audiometry.

- Tone audiometry in the sound field measurement is performed with warbler tones or narrowband noise with and without the sound processor.
- Speech audiometry is performed with speech in noise with S/N + 4 dB and a speech level of 65 dB or maximum speech perception of, for example, 65 dB with and without a sound processor.

The subjective experience is evaluated with questionnaires such as Glasgow Benefit Inventory (GBI), Abbreviated Profile of Hearing Aid Benefit (APHAB), The Speech, Spatial and Qualities of Hearing Scale (SSQ) or International Outcome Inventory for Hearing Aids (IOI-HA).^14^ At each follow-up visit, the magnetic strength should be checked. Evaluation of complications can be performed with the Holgers index for percutaneous BCDs and the IPS scale for all BCDs.^22^ The IPS scale is relatively new and is mostly used clinically but not regularly in research at this stage.^23^

## Conclusion

The development of hearing implants is fast, and many different options are available on the market. It is important that the healthcare staff is up to date to be able to inform the patients so that they can participate and make well-founded decisions about their hearing rehabilitation. Impartial evaluation of new and existing implants is extremely important for today's and tomorrow's patients as well as for optimizing healthcare resources. Today, objective data-driven decision-making is lacking and driving this forward should be a priority. This summary information is intended to be an unbiased resource for clinicians and provide an overview of available BCDs and AMEIs.
